# Epidemiological burden of schistosomiasis among schoolchildren in conflict-stricken mesoendemic districts of Yemen: A decade after national mapping

**DOI:** 10.1371/journal.pntd.0013723

**Published:** 2025-11-11

**Authors:** Walid M. S. Al-Murisi, Yahia A. Raja’a, Abdulsalam M. Al-Mekhlafi, Rashad Abdul-Ghani, Majid A. Al Samawi

**Affiliations:** 1 Department of Medical Parasitology, Faculty of Medicine and Health Sciences, Sana’a University, Sana’a, Yemen; 2 Department of Community Medicine, Faculty of Medicine and Health Sciences, Sana’a University, Sana’a, Yemen; 3 Faculty of Medicine and Health Sciences, Tropical Disease Research Center, University of Science and Technology (USTY), Sana’a, Yemen; Swiss Tropical and Public Health Institute: Schweizerisches Tropen- und Public Health-Institut, SWITZERLAND

## Abstract

**Background:**

Despite multiple rounds of mass drug administration (MDA), schistosomiasis remains a major public health problem in Yemen. This study assessed the burden of schistosomiasis among schoolchildren in mesoendemic districts over a decade after the 2014 national mapping in the context of the ongoing humanitarian crisis, conflict, and disrupted control efforts.

**Methods:**

A cross-sectional study was conducted among 348 schoolchildren aged 5–15 years in three randomly selected mesoendemic districts: Al Husha, Bura, and Habur Zulaymah districts. Data on sociodemographics and potential risk factors were collected using a pilot-tested, structured questionnaire. Parasitological examinations for *Schistosoma haematobium* and *S. mansoni* were performed using urine filtration and Kato-Katz techniques, respectively. Independent predictors of infection were identified using multivariable binary logistic regression.

**Results:**

The overall schistosomiasis prevalence among schoolchildren was 21% (95% CI: 17, 25), with 13.5% infected with *S. haematobium*, 6% with *S. mansoni*, and 1.4% co-infected. Compared to 2014, prevalence increased across all study districts, particularly for *S. haematobium*. Most infections were of light intensity. Infection with *S. haematobium* was significantly associated with dysuria, macrohematuria, microhematuria, and proteinuria. Independent predictors of schistosomiasis included male gender (AOR = 2.6; 95% CI: 1.34, 4.82; *P* = 0.003), age ≥ 10 years (AOR = 2.4; 95% CI: 1.20, 4.92; *P* = 0.013), household size larger than eight members (AOR = 2.4; 95% CI: 1.28, 4.63; *P* = 0.007), and contact with open water sources (AOR = 2.5; 95% CI: 1.20, 5.19; *P* = 0.014).

**Conclusion:**

Despite multiple MDA campaigns, schistosomiasis remains moderately endemic in the districts of Yemen classified as mesoendemic in 2014, with an increased *S. haematobium* prevalence. While MDA has reduced the prevalence of heavy infections, transmission persists. These findings underscore the need for an integrated strategy combining biannual MDA, health education, improved sanitation, and environmental management to interrupt transmission.

## Author summary

Even after repeated mass drug administration campaigns to combat schistosomiasis in Yemen, the disease is still a public health challenge. We conducted a survey among 348 schoolchildren in three randomly selected districts of the country to assess the prevalence, intensity, and risk factors associated with schistosomiasis over a decade since the national mapping in 2014 to see whether the situation has improved. We found that 21% of children were infected, with urogenital disease being more common than intestinal disease. However, most infections were mild. Notably, the disease prevalence increased in all districts compared to 2014, indicating persistent transmission. Our findings suggest that better access to safe water, proper sanitation and health education, along with regular treatment, are needed to stop the transmission of schistosomiasis in Yemen.

## Introduction

Schistosomiasis, a neglected tropical disease (NTD) caused by flukes of the genus *Schistosoma*, primarily affects populations living in tropical and subtropical regions [1]. It results in considerable morbidity and mortality, accounting for an estimated burden of 1.4 million disability-adjusted life years (DALYs) worldwide [2]. *Schistosoma* species of major public health concern include *S. haematobium* that causes urogenital schistosomiasis, and *S. mansoni* and *S. japonicum* that cause intestinal schistosomiasis [3]. Schoolchildren in low-income countries are particularly prone to high prevalence and intensity of schistosomiasis due to their more frequent exposure to infection [1,4].

Yemen is a low-income country, with the majority of its population living below the poverty line due to prolonged conflict, humanitarian crises, and limited access to safe water [5,6]. Schistosomiasis in the country is caused by *S. mansoni* and *S. haematobium*. In 2009, Yemen launched a nationwide elimination project for schistosomiasis and soil-transmitted helminths (STHs) [7]. The project involved implementing mass drug administration (MDA) with praziquantel (PZQ) and albendazole to all at-risk individuals [7]. Despite the extensive and dedicated efforts of Yemen’s National Schistosomiasis Control Programme (NSCP), schistosomiasis continued to be a public health problem in the country. In 2014, a nationwide mapping survey targeting school-aged children (SAC) found that schistosomiasis was endemic in 63.3% of districts, with more districts impacted by intestinal than urogenital schistosomiasis (54.2% *vs.* 31.6%) [8]. However, the risk of schistosomiasis among SAC in most districts was low (prevalence <10%) to moderate (prevalence ≥10% to <50%), with no district found to be at high risk of any type of schistosomiasis [8].

Since the baseline mapping in 2014, several sub-national studies across endemic areas of the country have shown an increase in schistosomiasis prevalence. For example, the prevalence of S. *mansoni* among schoolchildren in Al-Haimah AL-Dakheliah District of Sana’a Governorate increased sharply from 0.7% in 2014 to 33.9% in 2018, indicating a shift from low to moderate risk [9]. Meanwhile, the risk of schistosomiasis in Bani Matar District in the same governorate also shifted from non-endemic to low (prevalence of 1.4%) [9]. Likewise, *S. haematobium* prevalence among rural schoolchildren in Kharif District of Amran Governorate rose from 8.3% in 2014 to 34.8% in 2021 [10]. In Amd District of Hadhramaut Governorate in eastern Yemen, the prevalence of *Schistosoma* species among schoolchildren increased to 33.7%, indicating a shift from hypo- to mesoendemic risk [11]. In contrast, the prevalence of *S. mansoni* in An-Nadirah District of Ibb Governorate remained unchanged in 2019 compared to 2014 [12].

The limited number of recent studies on schistosomiasis across Yemen has hampered the availability of updated epidemiological data. Without these data, it becomes challenging to accurately assess the current burden of infection, monitor the effectiveness of control interventions, or identify emerging hotspots. Moreover, the ongoing conflict and humanitarian crisis since the nationwide mapping have likely disrupted NTD control programmes, including NSCP. Prolonged instability may interrupt or delay MDA campaigns, which are essential for reducing disease transmission and morbidity. Therefore, this study aimed to assess the burden of schistosomiasis among schoolchildren in mesoendemic communities in Yemen, with a focus on prevalence, intensity, and factors associated with infection a decade after national mapping and conflict.

## Methods

### Study design and population

A cross-sectional study was conducted among schoolchildren aged 5–15 years in three districts mesoendemic for schistosomiasis in Yemen.

### Study area

**Fig 1:**
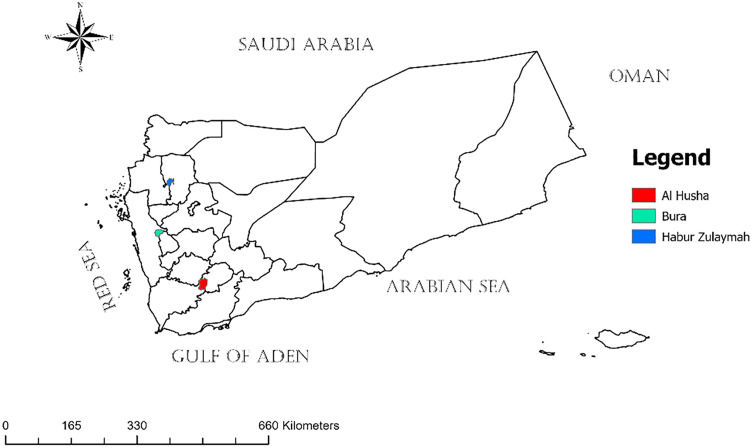
Map of Yemen showing the study districts. The basemap shapefile of Yemen’s administrative boundaries was obtained from the Global Administrative Areas (GADM, https://gadm.org/; CC BY 4.0 license). The map was created by the authors using ArcGIS 10.8 software.

Out of 25 districts previously classified as mesoendemic for schistosomiasis in Yemen based on the 2014 national mapping [13], three districts (Fig 1) were randomly selected to assess the burden of schistosomiasis after a decade of MDA, considering geographic diversity across the country. These districts include Al Husha District in Al-Dhalea Governorate in southwestern Yemen near the central highlands (13°45′N, 44°35′E), with an area of 501 km^2^ and a population of over 106 thousand inhabitants [14]; Bura District in Al Hudaydah Governorate on the western coastal plain of Tihama (14°45′N, 43°20′E), with an area of 242 km^2^ and a population of over 77 thousand inhabitants [14]; and Habur Zulaymah District in Amran Governorate, northern Yemen (16°00′N, 43°50′E), with an area of 200 km^2^ and a population of over 59 thousand inhabitants [14].

### Sample size and sampling strategy

A sample size of 318 schoolchildren was calculated using OpenEpi version 3.01 (available at www.openepi.com), based on an anticipated prevalence of 14.7% from a 2024 study in rural communities [15], with a 95% level of confidence, a 5% marginal error, a design effect of 1.5, and a 10% expected non-response rate. Nevertheless, 348 children were enrolled in this study to increase the precision of the estimates.

A multi-stage cluster sampling method was employed. In the first stage, three mesoendemic districts were randomly selected based on 2014 mapping data. In each district, two primary schools were randomly selected using a computer-based random number generator from a complete list of schools, yielding a total of six schools. Then, children were randomly selected by simple random sampling from the list of eligible children in each school according to enrollment size, with approximately 58 children sampled per school. If a selected child or his/her legal guardian refused to participate, the next eligible child listed in the school record was invited.

### Data and sample collection

A pre-tested questionnaire was used to collect data about children’s sociodemographic characteristics, hygiene practices, environmental sanitation, behaviors associated with an increased risk of infection, history of infection, and previous intake of PZQ. From February to May 2025, data were collected through interviews with the children, with teachers assisting when necessary.

Urine and stool samples were collected from children into properly labeled containers between 10 a.m. and 2 p.m., when egg excretion is optimal, after providing them with clear instructions. Urine samples were initially assessed macroscopically for visible hematuria, then tested for microhematuria and proteinuria using test strips (UroColor 9, Standard Diagnostics Inc., South Korea), as per the manufacturer’s instructions.

### Parasitological examination

The urine filtration technique was applied to detect and estimate the number of *S. haematobium* eggs per 10 mL of urine (EP10mL) [16,17]. The intensity of infection was categorized as light (≤50 EP10mL) or heavy (>50 EP10mL) [18,19]. On the other hand, the Kato-Katz technique was used to detect and estimate the number of *S. mansoni* eggs per gram (EPG) [18,20], using two thick smears from each stool sample. The intensity of infection was classified as light (<100 EPG), moderate (100–399 EPG), or heavy (≥ 400 EPG) [18]. To ensure diagnostic accuracy, quality control procedures were implemented by a team of qualified laboratory technicians, and 10% of the slides were randomly chosen and re-examined by an experienced technologist blinded to the initial results [18].

### Data analysis

Data were analyzed using IBM SPSS Statistics, version 24 (IBM Corp., Armonk, NY, USA). The prevalence of each *Schistosoma* species was reported along with its 95% confidence interval (CI). According to prevalence, infection risk in the study districts was categorized as low (<10%), moderate (10% to <50%), or high (≥50%) [21]. Associations between schistosomiasis and independent variables were assessed using the chi-square or Fisher’s exact test, and odds ratios (ORs) with 95% CIs were reported. Variables found to be significantly associated with schistosomiasis in univariate analysis were included in a multivariable binary logistic regression model, and adjusted ORs (AORs) with their 95% CIs were reported. A *P*-value of < 0.05 was considered statistically significant.

### Ethical considerations

This research strategy was authorized by the Research Ethics Committee (REC) of the Faculty of Medicine and Health Sciences, Sana’a University, Sana’a, Yemen. As the study was carried out in schools where the children’s parents/legal guardians were not present, written parental informed consent was waived by the REC because the study was conducted within school settings under the supervision of school authorities; however, they were informed about the study and their right to decline their children’s participation. Instead, permission was obtained from the head of each school after explaining the significance of the study. Only children who gave assent to participate were included in the study after the objective of their participation was explained in a way that they could understand. The privacy of children and the confidentiality of data were assured. Children found to be infected with schistosomiasis received appropriate treatment with PZQ after the approval of their guardians.

## Results

### Characteristics of children

The mean age of children was 10.3 ± 2.5 years (range: 5–15). Gender distribution was nearly equal (51.1% females *vs.* 48.9% males). More than half of the children’s fathers were educated (56.9%) compared to only 26.7% of their mothers. More than one-third of children’s fathers were employed (36.7%) compared to only 5.7% of their mothers. The mean household size was 7 ± 2.5 members, with most households comprising eight or fewer members (67.8%) (Table 1).

**Table 1:** Characteristics of schoolchildren included in the study (*N* = 348).

Characteristics	*n* (%)
**Gender**	
Male	170 (48.9)
Female	178(51.1)
**Age** (years) Mean ± SD: 10.3 ± 2.5 (range: 5–15)	
< 10	149 (42.8)
≥ 10	199 (57.2)
**Fathers’ education level**	
Educated^*^	198 (56.9)
Non-educated	150 (43.1)
**Mothers’ education level**	
Educated^*^	93 (26.7)
Non-educated	255 (73.3)
**Fathers’ employment status** ^**#**^	
Employed	126 (36.7)
Unemployed	217 (63.3)
**Mothers’ employment status**	
Employed	20 (5.7)
Unemployed	328 (94.3)
**Household size** (members) Mean ± SD: 7 ± 2.5 (range: 3–15)	
≤ 8	236 (67.8)
> 8	112 (32.2)

* Educated (at least primary school certificate). ^#^ Five missing cases.

### Prevalence of schistosomiasis among schoolchildren

The overall prevalence of schistosomiasis among schoolchildren in the three study districts was 21% (73/348; 95% CI: 17, 25). Among the infected children, 47 (13.5%) had *S. haematobium*, 21 (6%) had *S. mansoni*, and 5 (1.4%) were co-infected with both species. Table 2 shows that the prevalence was higher in 2025 compared to 2014 in the districts of Al Husha (20.5% *vs.* 18.8%), Bura (14.3% *vs.* 11.6%), and Habur Zulaymah (24.2% *vs.* 11.8%). *S. haematobium* prevalence increased by over threefold in Al Husha district and by twofold in Bura and Habur Zulaymah districts in 2025 compared to 2014, while *S. mansoni* prevalence declined by one-third in Al Husha and by half in Habur Zulaymah district.

**Table 2:** District-level schistosomiasis prevalence in 2025 compared to the 2014 national mapping.

District	Survey year	Prevalence (%)		
		Any *Schistosoma* species	*S. mansoni*	*S. haematobium*
Al Husha^a^	2014	18.8	17.2	3.6
	2025	20.5	11.6	11.6
Bura^b^	2014	11.6	9.2	2.8
	2025	14.3	10.0	5.7
Habur Zulaymah	2014	11.8	2.0	10.2
	2025	24.2	0.8	23.5

^a^2.7% were co-infection with both species; ^b^ 1.4% were co-infection with both species.

In terms of infection intensity, most cases fall within the light intensity category; only 5.2% of *S. haematobium* infections and 0.3% of *S. mansoni* infections were classified as heavy intensities (Table 3).

**Table 3:** Intensity of schistosomiasis among infected schoolchildren in the study districts.

Intensity category	Type of infection			
	*S. haematobium* (*N* = 52)		*S. mansoni* (*N* = 26)	
	*n* (%)	Mean EP10mL (95% CI)	*n* (%)	Mean EPG (95% CI)
Light	34 (65.4)	15.7 (10.9, 20.4)	22 (84.6)	52.4 (41.2, 63.6)
Moderate	0 (0.0)	NA	3 (11.5)	128 (93.6, 162.4)
Heavy	18 (34.6)	138.9 (101.0, 176.8)	1 (3.8)	480 NA

**EP10mL:** eggs per 10 mL of urine; **EPG:** eggs per gram of stool; **CI:** confidence interval; **NA:** not applicable.

### Clinical indicators of schistosomiasis

There was a statistically significant association between *S. haematobium* infection among schoolchildren and the presence of dysuria (*P* < 0.001), macrohematuria (*P* < 0.001), microhematuria (*P* < 0.001), and proteinuria (*P* = 0.001). Conversely, *S. mansoni* infection was not significantly associated with hematochezia (*P* = 1.000) or diarrhea (*P* = 0.244) (Table 4).

**Table 4:** Clinical indicators of schistosomiasis among schoolchildren in previously mesoendemic districts of Yemen (2025).

Indicator	*N*	Infection rate	*P*-value
		*n* (%)	
***S. haematobium* infection**			
**Dysuria**			
No	267	28 (10.5)	<0.001
Yes	81	24 (29.6)	
**Macrohematuria**			
No	333	44 (13.2)	<0.001
Yes	15	8 (53.3)	
**Microhematuria**			
Negative	304	24 (7.9)	<0.001
Positive	44	28 (63.6)	
**Proteinuria**			
Negative	290	35 (12.1)	0.001
Positive	58	17 (29.3)	
***S. mansoni* infection**			
**Hematochezia**			
No	345	26 (7.5)	1.000
Yes	3	0 (0.0)	
**Diarrhea**			
No	321	26 (8.1)	0.244
Yes	27	0 (0.0)	

***N:*** total number of children examined; ***n:*** number of children infected with the corresponding species.

### Sociodemographic factors associated with schistosomiasis

Univariate analysis shows that male gender (OR = 1.9, 95% CI: 1.14, 3.27; *P* = 0.014), age of ≥10 years (OR = 4.1, 95% CI: 2.17, 7.62; *P* < 0.001) and households with more than eight members (OR = 2.4, 95% CI: 1.40, 4.02; *P* = 0.001) were significantly associated with a higher risk of schistosomiasis compared to their counterparts. In contrast, there was no significant association between schistosomiasis and fathers’ education or parental employment status (Table 5).

**Table 5:** Sociodemographic factors associated with schistosomiasis among schoolchildren in previously mesoendemic districts of Yemen (2025).

Variables	*N*	Infection with any *Schistosoma* species		*P*-value
		*n* (%)	OR (95%CI)	
**Gender**				
Female	178	28 (15.7)	Reference	0.014
Male	170	45 (26.5)	1.9 (1.14, 3.27)	
**Age** (years)				
< 10	149	14 (9.4)	Reference	<0.001
≥ 10	199	59 (29.6)	4.1 (2.17, 7.62)	
**Fathers’ education**				
Educated	93	20 (21.5)	Reference	0.884
Uneducated	255	53 (20.8)	1.0 (0.54, 1.71)	
**Fathers’ employment status**				
Employed	126	22 (17.5)	Reference	0.221
Unemployed	217	50 (23.0)	1.4 (0.81, 2.47)	
**Mothers’ employment status**				
Employed	20	4 (20.0)	Reference	1.000
Unemployed	328	69 (21.0)	1.1 (0.35, 3.29)	
**Household size** (members)				
≤ 8	236	38 (16.1)	Reference	0.001
> 8	112	35 (31.3)	2.4 (1.40, 4.02)	

***N*:** total number of children examined; ***n*:** number of children infected; **OR:** Odds ratio; **CI**: confidence interval.

### Behavioral and environmental factors associated with schistosomiasis

Contact with open water sources (OR = 3.8, 95% CI: 1.89, 7.46; *P* < 0.001), not wearing shoes before going outside (OR = 1.8, 95% CI: 1.00, 3.41; *P* = 0.048), and a history of schistosomiasis (OR = 3.1, 95% CI: 1.81, 5.31; *P* < 0.001) were significantly associated with a higher risk of infection among children (Table 6).

**Table 6:** Behavioral and environmental factors associated with schistosomiasis among schoolchildren in previously mesoendemic districts oF Yemen (2025).

Variables	*N*	Infection with any *Schistosoma* species		*P*-value
		*n* (%)	OR (95% CI)	
**Contact with open water sources**				
No	121	11 (9.1)	Reference	<0.001
Yes	227	62 (27.3)	3.8 (1.89, 7.46)	
**Wearing shoes before going outside**				
Yes	285	54 (18.9)	Reference	0.048
No	63	19 (30.2)	1.8 (1.00, 3.41)	
**Availability of a household toilet**				
Available	335	69 (20.6)	Reference	0.484
Not available	13	4 (30.8)	1.7 (.51, 5.73)	
**Availability of a school toilet**				
Available	271	58 (21.4)	Reference	0.715
Not available	77	15 (19.5)	0.9 (0.47, 1.68)	
**Household water source**				
Piped	256	55 (21.5)	Reference	0.698
Unpiped	92	18 (19.6)	0.9 (0.49, 1.61)	
**Home sanitation** ^a^				
Improved	154	34 (22.1)	Reference	0.653
Unimproved	194	39 (20.1)	0.9 (0.53, 1.49)	
**History of schistosomiasis**				
No	250	38 (15.2)	Reference	<0.001
Yes	98	35 (35.7)	3.1 (1.81, 5.31)	
**Previous PZQ intake**				
Yes	159	27 (17.0)	Reference	0.093
No	189	46 (24.3)	1.6 (0.93, 2.67)	

***N*:** number of children examined; ***n*:** number of children infected; **OR:** odds ratio; **CI:** confidence interval; **PZQ:** praziquantel. ^a^ Improved sanitation was defined as flush or pour flush toilet connected to a piped sewer system or pit latrine, while unimproved sanitation referred to the absence of a toilet or the use of a flush or pour flush toilet that discharges into an open area.

### Independent predictors of schistosomiasis

Multivariable binary logistic regression analysis shows that male gender (AOR = 2.6; 95% CI: 1.34, 4.82; *P* = 0.003), age ≥ 10 years (AOR = 2.4; 95% CI: 1.20, 4.92; *P* = 0.013), household size of more than eight members (AOR = 2.4; 95% CI: 1.28, 4.63; *P* = 0.007), and contact with open water sources (AOR = 2.5; 95% CI: 1.20, 5.19; *P* = 0.014) were independent predictors of infection among children (Table 7).

**Table 7:** Independent predictors of schistosomiasis among schoolchildren in previously mesoendemic districts in Yemen (2025).

Predictor	AOR (95% CI)	*P-*value
Male gender	2.6 (1.38, 4.75)	0.003
Age ≥ 10 years	2.6 (1.29, 5.01)	0.007
Family size > 8 members	2.4 (1.31, 4.51)	0.005
Contact with open water sources	2.5 (1.20, 5.19)	0.014

**AOR:** adjusted OR; **CI:** confidence interval.

## Discussion

Despite the implementation of multiple rounds of MDA in Yemen since the 2014 national mapping, schistosomiasis remains a major concern in districts that were previously classified as mesoendemic. In the present study, the overall prevalence among schoolchildren across the three surveyed districts was 21%, with *S. haematobium* (14.9%) being more prevalent than *S. mansoni* (7.5%). This finding indicates that the study districts still fall within the WHO’s classification for moderate endemicity [21]. Of particular concern is the observed increase in *S. haematobium* prevalence across all districts compared to baseline levels in 2014, in comparison to the declining prevalence of *S. mansoni*. This shift in species prevalence reflects a shift in transmission dynamics, which may be attributed to such factors as ecological change, water contact behavior, and snail distribution. The findings of this study are consistent with previous studies on schistosomiasis prevalence among rural children in Yemen. For example, a large-scale survey across five governorates (Dhamar, Hodeidah, Ibb, Sana’a, and Taiz) reported an overall prevalence of 31.8% among children [22]. Similarly, a study in Shara’b Al-Raona district of Taiz found that 24.6% of schoolchildren were infected with *Schistosoma* species [23]. MDA alone has not been sufficient to interrupt transmission in these mesoendemic districts, particularly under the challenges posed by prolonged conflict, weakened health systems, and limited access to safe water and sanitary facilities. The lack of complementary interventions, such as health education, snail control and environmental management, reduces the long-term effectiveness of MDA [24], highlighting the need for an integrated control approach. Moreover, reinfection remains a continual challenge in endemic areas following preventive chemotherapy with PZQ [25–27].

The prevalence of *S. haematobium* observed in the present study (14.9%) aligns closely with the prevalence of 14.7% recently reported among SAC in rural areas of Hadhramout Governorate, east of the country [15]. Similarly, 18.1% of children in the southern governorate of Abyan were found to be infected with *S. haematobium* [28]. A notably higher prevalence of *S. haematobium* has been recently documented among SAC in Amd district of Hadhramout and Kharif district of Amran, with rates of 33.7% and 34.8%, respectively [10,11]. In contrast, a lower prevalence of *S. haematobium* was reported among children from the governorates of Taiz (7.4%) [29], Sa’adah (3.3%) [30], and Hajjah (1.7%) [31]. These findings highlight significant heterogeneity in disease burden in different districts, which may reflect local ecological and behavioral factors, such as proximity to infested water sources, frequency of water contact, and variations in snail host density. On the other hand, the prevalence of *S. mansoni* observed in the present study (7.5%) is comparable to that reported from Ibb Governorate (6.5%) [12] and a multi-governorate survey across five governorates of the country (9.3%) [22]. However, it is lower than the prevalence reported in other districts in the governorates of Sana’a (33.9%) [9], Taiz (20.8%) [29], and Taiz (14.3%) [32]. Conversely, lower rates have been documented in Bani Matar district of Sana’a Governorate (1.4%) [9] and in parts of Sa’adah Governorate (1.1%) [30]. The findings of the present study, along with previous ones, highlight the geographically heterogeneous nature of schistosomiasis in Yemen, emphasizing the need for subnational mapping and district-specific interventions.

The majority of *S. haematobium* and *S. mansoni* infections among schoolchildren in this study were of light intensity, with heavy infections being infrequent (34.6% and 3.8%, respectively). This pattern is likely to reflect the positive impact of previous MDA campaigns in reducing the prevalence of high-intensity infections, which are more strongly linked to disease-related morbidity. Similar patterns have been reported in other MDA-targeted districts across the country [9–12]. It is worth noting that the 2014 national mapping did not estimate the intensity of *Schistosoma* infections due to time and logistical constraints [13], leaving a gap in the baseline data to assess the change in infection intensity in the study districts. In this context, this study provides valuable baseline data that can support monitoring and assessment of ongoing and future control efforts. Overall, the low prevalence of heavy-intensity infections observed in this study supports the role of school-based chemotherapy in reducing disease burden but also underlines the need for sustained integrated control measures to eliminate the disease.

Self-reported dysuria, visible hematuria, microhematuria, and proteinuria showed a significant association with *S. haematobium* infection, indicating their usefulness as sensitive indicators for field surveys, as previously documented in studies from Yemen [11,28,33]. The lack of a significant association between *S. mansoni* infection and diarrhea or hematochezia is probably attributed to the predominance of light-intensity infections in the study population, which is consistent with findings elsewhere [34]. Nevertheless, it is important to recognize that chronic low-intensity infections can still contribute to long-term morbidity [35–37].

The demographic and behavioral risk factors identified in this study align with previous studies in Yemen. Male gender, age of 10 years and older, larger household size, and exposure to open water sources were all recognized as predictors of schistosomiasis in this study, aligning with predictors identified in previous studies in the country [9,10,22] and are supported by a systematic review and meta-analysis conducted in endemic African countries [38]. These findings highlight the role of gender, age, household crowding, and water-related behaviors in sustaining transmission. Notably, parental illiteracy and lack of access to safe water, previously identified as risk factors for infection elsewhere in the country [9,22,33], did not show a significant association with schistosomiasis in the present study. This discrepancy may be partly attributed to the homogeneously low socioeconomic status of the study population, where poor educational levels and insufficient access to clean water are widespread and thus offer limited variability for detecting statistical associations. Although univariate analysis showed that a history of previous infection was significantly associated with a higher risk of infection, this association did not remain significant after adjusting for other factors in the multivariable model. This finding suggests that its effect may be confounded or mediated by other variables.

This study provides timely and policy-relevant epidemiological insight into schistosomiasis in mesoendemic districts of Yemen. Nevertheless, several limitations should be considered. Although the selected districts aimed to capture geographic diversity, the results may not be representative of all mesoendemic areas in the country, highlighting the need for broader studies to better understand national transmission patterns. Additionally, the cross-sectional design limits the ability to track changes in disease dynamics over time, underscoring the importance of longitudinal research to evaluate the long-term effects of MDA interventions. The use of self-reported behavioral data may also be subject to recall or social desirability bias. Moreover, the lack of baseline infection intensity data from the 2014 national mapping hampers direct comparisons of disease burden beyond prevalence estimates. Because the raw data from the 2014 national mapping were unavailable, it was not possible to perform a rigorous statistical comparison between the baseline and current prevalence estimates, and descriptive comparisons were therefore employed to illustrate changes in schistosomiasis transmission patterns.

## Conclusion

Schistosomiasis persists as a public health problem with a moderate risk in mesoendemic districts of the country, with an increase in the prevalence of *S. haematobium* compared to baseline data in 2014. The majority of infections were of light intensity, indicating a positive impact of MDA campaigns in reducing the level of morbidity but not interrupting the ongoing transmission. These findings highlight the limitations of chemotherapy alone and emphasize the need for a comprehensive, multisectoral control strategy that integrates biannual MDA for all SAC, health education, improved sanitation, and water resource management to reduce the disease burden and prevent reinfection. In alignment with WHO’s guidelines, schistosomiasis test-and-treat services should be also integrated into routine primary healthcare.

## Supporting information

S1 Data Complete minimal dataset of this study. (XLSX)

S1 File Questionnaire. Data collection questionnaire for this study.
(DOCX)
