# Supplementary material for: Epidemiological burden of schistosomiasis among schoolchildren in conflict-stricken mesoendemic districts of Yemen: A decade after national mapping
Source: PLoS Negl Trop Dis. 2025 Nov 11;19(11):e0013723. doi: 10.1371/journal.pntd.0013723 (PMC12617871; doi:10.1371/journal.pntd.0013723)
Supplement: S1 File — Data collection questionnaire for this study. (DOCX) [file pntd.0013723.s002.docx]

**Questionnaire (English Version)**

**Study title: Epidemiological burden of schistosomiasis among schoolchildren in conflict-stricken mesoendemic districts of Yemen: A decade after national mapping.**

Use ( √ ) mark for student choice

School name: _________________ Date of Interview: __/__/_____

**Section 1. Demographic and sociodemographic data:**

ID No.:­­_______________ Class:___________ Village name: _____________

Gender: Male: ____ Female: ____

Age (years/ months): ­­­­­­­­­­ ____ ____

Father’s education Eeducated ____ illiterate ____ education level:_______ Mother’s education: Eeducate ____ illiterate ____ education level:_________ Father’s employment status:

Not working ____ Employee ___ Farmer: ____ Other(specify):__________ Mother’s employment status:

Not working ____ Employee ___ Farmer: ____ Other(specify):__________

Household size (members): ____

**Section 2: Environmental data:**

Presence of latrines in house Yes ____ No ____

Presence of latrines in school Yes ____ No ____

Where is your bathroom water discharge? Sewage ____ Irrigation ____ outside ____

Source of drinking water in the house:

Unpiped (stream, rain, well, etc.) ____

Piped: ____

What is the type of water source?

Dam ____ stream ____ pond ____ spring ____ others ………………

What is the source of drinking water in the school?

unpiped source (stream, rain, well, etc.): ___

Piped source: ____

What is the type of water source?

Dam _____ ____ stream ____ pond ____ spring ____ other (specify)___________

**Section 3: Practices and other predictor data:**

Did you have blood in your urine in the past two weeks? Yes ____ No ____

Did you experience dysuria in the past two weeks? Yes ____ No ____

Did you have blood in your stool in the past two weeks? Yes ____ No ____

Did you have diarrhea in the past two weeks? Yes ____ No ____

Were you diagnosed with bilharziasis previously? Yes ____ No ____

*If yes,*

Did you take anti-schistosome treatment? Yes ____ No ____

Do you come in contact with water sources outside the house? Yes ____ No ____

*If yes,*

How often do you come into contact with open water each week?
☐ Daily
☐ 2–3 times a week
☐ Less than once a week
☐ I do not come into contact with open water

For what reasons do you come in contact with open-source water outside the house (*tick all that apply*!)?

Swimming Yes ____ No ____

Bathing Yes ____ No ___

Washing clothes Yes ____ No ____

Wading or walking through the stream Yes ____ No ____

Farming Yes ____ No ____

Do you urinate in open water sources outside the house? Yes ____ No ____

Do you defecate in open water sources outside the house? Yes ____ No ____

Do you wear shoes when going outside the house? Yes ____ No ____

Do you know that schistosomiasis can be transmitted through contact with contaminated water? Yes ____ No ___

**Section 4: Parasitological and laboratory data:** *(to be filled following examination of samples)*

**Stool findings:**

- Blood:______ Mucus:_______

- Presence of *S. mansoni*: Yes ____ No ***____***

*If “yes”,* EPG*= _________.*

- Other parasite species identified: ……………………………………………………………

**Urine findings:**

- Visible hematuria

Positive ____ Negative __ _

- Microhematuria by strip

Positive ____ Negative __ _

- ­­­­­­­­­­­­­­Proteinuria by strip

Positive ____ Negative __ _

- Presence of *S. haematobium*

Yes ____ No ***____***

*If “yes”, eggs/ 10 ml= _______.*

- Other parasite species identified …………………………………………
